# Systematic computerised cardiovascular health screening for people with severe mental illness

**DOI:** 10.1192/pb.bp.113.045955

**Published:** 2014-12

**Authors:** David Yeomans, Kate Dale, Kate Beedle

**Affiliations:** 1 Leeds and York Partnership NHS Foundation Trust; 2 Bradford District NHS Care Trust; 3 NHS West and South Yorkshire and Bassetlaw Commissioning Support Unit

## Abstract

**Aims and method** People with severe mental illness (SMI) die relatively young, with mortality rates four times higher than average, mainly from natural causes, including heart disease. We developed a computer-based physical health screening template for use with primary care information systems and evaluated its introduction across a whole city against standards recommended by the National Institute for Health and Care Excellence for physical health and cardiovascular risk screening.

**Results** A significant proportion of SMI patients were excluded from the SMI register and only a third of people on the register had an annual physical health check recorded. The screening template was taken up by 75% of GP practices and was associated with better quality screening than usual care, doubling the rate of cardiovascular risk recording and the early detection of high cardiovascular risk.

**Clinical implications** A computerised annual physical health screening template can be introduced to clinical information systems to improve quality of care.

People with a diagnosis of severe mental illness (SMI) such as schizophrenia and bipolar disorder die 15–20 years earlier than the general population, mainly from natural causes.^1^ In particular, they have an increased risk of cardiovascular disease.^2^ This health inequality was reviewed by the Disability Rights Commission in a 2006 report titled *Equal Treatment: Closing the Gap*.^3^ Deprivation and lifestyle were major factors, but not sufficient to account for the health inequalities. The report proposed that ‘diagnostic overshadowing’, or clinical blindness to physical problems in people with mental illness, was a form of inadvertent discrimination by health professionals that led to underdiagnosis, underinvestigation and undertreatment of potentially preventable or treatable physical disease in people with mental illness. The Royal College of Psychiatrists has made recommendations to address physical health inequalities through better training of psychiatrists and better collaboration with primary care.^4^ Psychiatrists believe that physical health is important and are aware that pharmacological treatment is another factor producing a higher risk of mortality. Antipsychotic medications can cause sudden cardiac death^5^ and diabetes,^6^ and have a dose-dependent relationship to mortality.^7^

Early death in people with SMI has been recognised since the 1990s.^8,9^ Since then evidence has grown that there are high death rates from cardiovascular disease and other natural causes.^10–13^ The risk of dying from cardiovascular disease alone significantly exceeds the risk of dying from suicide.^12,14^ In contrast to suicide risk assessment and prevention, cardiovascular risk assessment is relatively well evidenced, with clinical algorithms for cardiovascular risk prediction and a range of clinical interventions for primary prevention, such as lifestyle advice and treatment for elevated blood pressure and lipids. However, routine screening for cardiovascular risk is less common than screening for suicide risk, especially in secondary care. All people diagnosed with an SMI such as schizophrenia should have an annual physical health check that includes metabolic screening.^15^ The National Institute for Health and Care Excellence (NICE) has also recommended a standard cardiovascular disease risk calculation as part of the annual health check since 2002.^16^ In this study we focus on the cardiovascular risk assessment element of the computerised physical health check template.

## Aims

We planned to carry out a cross-sectional retrospective service evaluation of the quality of physical health monitoring of all registered SMI patients in the Bradford and Airedale region using the standards recommended by NICE for schizophrenia.^17^ We designed and implemented a computer template for the primary care information system to support a standard annual physical health check for SMI patients. We wanted to see whether patients who received the template-based screening got better or worse quality care than patients who did not.

## Method

All but one general practice in the Bradford region used the same computer system, SystmOne (www.tpp-uk.com/modules), allowing data on almost the whole SMI register to be anonymously and centrally collated. This would have been a huge task if done manually using paper-based checklists.

We designed a physical health screening template for the primary care computer system to help general practitioners (GPs) carry out a high-quality annual health check using standards recommended by NICE for physical health checks in schizophrenia.^17^ We designed it to help GPs submit data returns for the Quality and Outcomes Framework (QOF)^18^ which makes payments to GP practices for specific tasks, including physical health monitoring in SMI.

The physical health screening template is two pages long (with two further pages of explanation and information). It is updated by the data quality team if NICE standards and QOF criteria change. It guides GPs to collect the clinical information needed to identify a range of physical morbidity and health risks, including cardiovascular risk, without needing to learn the detailed NICE guidance or the requirements of QOF. The template looks like every other template on the system and fits into GPs’ normal workflows. It automatically includes any pre-existing data from the patient record in order to increase efficiency. It facilitates the allocation of tasks to the primary care team (e.g. ordering blood tests). Results are fed back through the usual channels in the computer system. This integrates physical health monitoring for SMI patients into normal practice.

We then began a process of promoting the template to GP practices in 2011–2012. All 80 practices using SystmOne were contacted and 48 received a 30-minute staff training session delivered by the data quality specialist (K.B.) and/or the physical health project lead (K.D.). Primary care teams decided if and when to use the template.

We carried out the evaluation of template use retrospectively, in a naturalistic setting, using data that were recorded in the course of day-to-day practice by primary care teams in the year leading up to the assessment date, 1 July 2013. We used CTV3 (Clinical Terms Version 3) Read codes (http://systems.hscic.gov.uk/data/uktc/readcodes/index_html), including codes used in the QOF codes formulary, to construct database reports on template usage. CTV3 Read codes identify elements of activity in the primary care information system and QOF codes are used to generate incentive payments to GP practices to improve service quality. There are specific codes for physical health monitoring in SMI and also for the details of clinical historical data, examination findings and test results. We wrote our reports in the SystmOne reporting module. Almost all practice activity is recorded on the computer system, and our method necessarily disregards any activity not recorded in this way.

The reports captured activity for all patients registered with SystmOne GPs in the Bradford and Airedale region. We compared the usual practice of annual monitoring of physical health of SMI patients in primary care with the new practice of using a standard physical health screening template in the annual check-up.

We chose to use the standard QRisk®2 cardiovascular disease risk calculator (http://qrisk.org/) in our template. The information system already had rules that calculated ‘default’ QRisk®2 scores even without health data entered: average population data are inserted into blank fields within the default QRisk®2 calculator, meaning that the scores potentially underrepresent risk in a high-risk population such as SMI patients. We created a new report to identify ‘data-rich’ QRisk®2 scores in which the following four factors were always recorded: systolic blood pressure, HDL:cholesterol ratio, smoking status and body mass index. In doing this we aimed to audit calculations that were more accurate than those provided as a default by the computer system.

We were aware that there was a second Joint British Societies CVD risk calculator (http://www.jbs3risk.com) available to GPs on the system and recorded when it was used.

We had support from a number of primary care leaders, including nurses and doctors. We also had support from the mental health services in Bradford District NHS Care Trust and the NHS West and South Yorkshire and Bassetlaw Commissioning Support Unit. Ethical boundaries on the use of ‘big data’ are not yet standardised and we thought it appropriate to have oversight for the project from the relevant employers. No patient identifiable data were used in the evaluation.

## Results

Results were derived from reports written for this evaluation in the SystmOne reports module. We used all relevant CTV3 Read codes and QOF codes recorded in the system to construct the reports. Tests for significance in our comparisons were calculated using the chi-square test function in Microsoft Excel. We examined two main areas: the uptake of the template following a single 30-minute promotion and differences in cardiovascular screening outcomes between patients who were and those who were not offered the template-based health check.

On 1 July 2013, there were 568 677 patients registered fully with GPs in Bradford and Airedale. There were 5056 people on the SMI register. The register was incomplete because 576 patients (10.2% of the potential register) were excluded after initial allocation for various reasons. This compares with only 3.3% exclusions of potential diabetic register patients in the region (*P*<0.01). Only 32% of people on the SMI register received an annual physical health check recorded by a QOF code.

Sixty general practices (75%) used the screening template at least once during the 12-month period from 1 July 2012; 12 of these had not received the direct health promotion session, but had discovered the template on the system independently and 20 practices included at least 10 patients.

Overall, 335 template-based physical health reviews were carried out, which amounted to 20.5% (335/1631) of patients given a physical health review in the 12-month period. Of those, 23% (77/335) had a ‘data-rich’ QRisk®2 recorded compared with only 8.5% (120/1296) of patients who had an annual physical review without a template-based health check (*P*<0.01) (Table 1).

QRisk®2 scores above 20% indicate a need for primary intervention even with overt pathology, because the risk of a fatal cardiovascular event within 10 years is significant. QRisk®2 scores greater than 20% were found in 3.9% of template-based reviews, compared with 1.5% of reviews not using the template (*P*<0.01). This difference is broadly in line with the increased proportion of ‘data-rich’ QRisk®2 scores associated with template-based reviews. This suggests that use of the template significantly increased the detection of cardiovascular risk, compared with usual practice, and that this may simply be a feature of screening patients more accurately by using a high standard of QRisk®2 measurement.

QRisk®2 scores greater than 20% were found in 16.7–16.9% of ‘data-rich’ QRisk®2 records, regardless of whether or not the template was used. This rate is somewhat higher than the 9.3% rate found in the general adult population in Bradford and Airedale derived from the GP database (*P*<0.05) and the 10.5% population estimate

**Table 1:** Comparison of QRisk®2 records and scores for template-based reviews and non-template-based reviews

	Template-based reviews			Non-template-based reviews		
	Number	Reviews	Data-rich QRisk^A^2 records	Number	Reviews	Data-rich QRisk®2 records
Reviews	335	-	-	1296	-	-
						
Data-rich QRisk®2 records	77	23.0%	-	120	8.5%	-
						
Data-rich QRisk®2 scores ≥20	13	3.9%	16.9%	20	1.5%	16.7%

from Dalton.^19^ This demonstrates how the health inequality detected in SMI research can also be found using a general practice database not designed for research.

Use of the annual physical health check template was associated with an increased proportion of patients receiving individual measures relevant to calculating cardiovascular risk (*P*<0.01). Table 2 compares the frequency of recorded measures that are used in the calculation of cardiovascular risk for the whole SMI register and for those patients who had a template-based review.

Clinical history and examination measures were conducted for about three-quarters of patients on the SMI register, but fewer than half had the necessary blood tests for lipids. By contrast, three-quarters of patients with a template-based review received the recommended lipid screening and over 90% had the history and physical examination measures. This suggests that use of the template had the effect of encouraging primary care teams to collect the data needed to make high-quality cardiovascular risk assessments.

## Discussion

Our data are derived from an administrative system rather than a research protocol and therefore rely on clinicians’ behaviour, the IT system architecture and reporting capabilities. SystmOne has a powerful reporting module that makes use of CTV3 Read codes and QOF codes. Reports can be built that accurately represent the physical health screening activity offered to patients. It is likely that all activity recorded on the system was captured and this accurately reflects the real rate of physical health checks recorded in primary care.

Based on our reports, we found that people with SMI experienced disadvantages in health screening compared with other high-risk groups. Fewer people with SMI were included in the SMI register compared with the proportion of people with diabetes (another high-risk group) included in the diabetes register. Despite long-standing evidence of high physical and cardiovascular health risks, SMI patients are less likely than patients with diabetes to have access to physical health checks in primary care. The death rate in adults with SMI is four times higher than in the general population^20^ and health screening is potentially life-saving in this high-risk group.

Although there are areas of good practice, the systematic prevention and treatment of physical disease in people with SMI has received relatively little attention. Many guidelines have been produced but none have been

**Table 2:** Comparison of health screening measures recorded for patients with serious mental illness with and without a health screening template

Proportion of patients with measures recorded	Whole SMI register	SMI register with template review
Systolic blood pressure	75%	97%
		
Body mass index	71%	91%
		
HDL:cholesterol ratio	45%	76%
		
Smoking status	72%	92%

HDL, high-density lipoprotein; SMI, serious mental illness.

adequately implemented.^21^ De Hert *et al*^22^ have helpfully summarised a range of actions that could be taken, but were unclear on the mechanism to bring about these quality improvements. Health screening using a paper-based template is one possible mechanism and has been promoted by the Royal College of Psychiatrists (using the Positive Cardiometabolic Health (Lester) Algorithm^23^) and Rethink, a campaigning mental health charity.^24,25^ It is hard to see how these screening templates can be systematically implemented in paper form. Our study took the extra step of integrating a standard health screening tool into the primary care information system, so it could be automated, in the hope that this would facilitate the practice of physical health screening in SMI.

Overall, we found that adherence to the NICE standard of one physical check-up per year for SMI patients was lamentably low at 32%. This could be due to low adherence to the standard for health checks or low adherence to recording them with the correct code. Cardiovascular risk assessment received a low priority, with less than 10% of patients on the SMI register getting a high-quality ‘data-rich’ risk calculation. If this is merely a data quality issue, then better recording would help. Our method depended on accurate data recording and could not tease out how much unrecorded activity may have taken place.

Uptake of the template was about 1 in 5 of all annual physical reviews, which is encouraging given that there was no incentive to use the template other than to improve quality of care. We did not employ any performance targets.

Use of the template was associated with more than double the rate of adherence to the NICE standards in relation to the calculation of cardiovascular risk. The template was also associated with more than double the rate of detection of significant cardiovascular risk. These findings suggest that, by making a computerised health screening tool available, GP teams were helped to carry out higher-quality physical health reviews and detect more patients at risk of early cardiovascular death. Conversely, our results also suggest that low-quality screening fails to identify cardiovascular risk. The use of automated QRisk®2 calculators that fill in empty fields with average data should be discouraged with SMI patients. It is possible, but unlikely, that GPs could have biased results by selecting high-risk patients for template-based reviews.

NICE and QOF have not yet delivered universal physical health checks for people with severe mental illness in primary care and additional approaches to improve practice are needed. Although our computer-based template seems to increase quality, it may not be easy to replicate this work in future, since the standard QOF incentive for annual health checks in primary care will be removed in 2014. Instead, NHS England will write a new CQUIN (Commissioning for Quality and Innovation) incentive that will encourage mental health trusts to monitor and improve the physical health of SMI patients.^26^ The problem with this secondary care approach is that mental health trusts lack the clinical skills in physical healthcare and the sophisticated information systems present in primary care. However, it should still be possible to introduce health screening templates into mental health information systems and build reports from them.

The computerised physical health screening template is a device that can facilitate high-quality practice. We found that practices that received promotion of the template were more likely to use it, so stronger promotion of a computerised physical health check template could increase the uptake. In secondary care trusts, a physical health screening template could be paired with performance targets to achieve the new CQUIN payment.

It is not yet clear whether screening for cardiovascular risk in people with SMI can lead to a reduction in early death, although structured intervention programmes based on screening have demonstrated small health gains.^27^ Long-term longitudinal studies will be needed to answer this question.

## References

[1] Thornicroft G Physical health disparities and mental illness: the scandal of premature mortality. Br J Psychiatry 2011; 199: 441–2. 22130744 10.1192/bjp.bp.111.092718

[2] Lambert T Newcomer J Are the cardiometabolic complications of schizophrenia still neglected? Barriers to care. Med J Australia 2009; 190 (suppl 4): 39–42. 10.5694/j.1326-5377.2009.tb02374.x 19220173

[3] Disability Rights Commission. Equal Treatment: Closing the Gap. A Formal Investigation into Physical Health Inequalities Experienced by People with Disabilities and/or Mental Health Problems. DRC, 2006 (http://disability-studies.leeds.ac.uk/files/library/DRC-Health-FI-main.pdf).

[4] Royal College of Psychiatrists. *Physical Health in Mental Health* (Occasional Paper OP67). Royal College of Psychiatrists, 2009.

[5] Ray A Chung C Murray K Hall K Stein C Atypical antipsychotic drugs and the risk of sudden cardiac death. N Engl J Med 2009; 360: 225–35. 19144938 10.1056/NEJMoa0806994 PMC2713724

[6] Kessing LV Thomsen AF Mogensen UB Andersen PK Treatment with antipsychotics and the risk of diabetes in clinical practice. Br J Psychiatry 2010; 197: 266–71. 20884948 10.1192/bjp.bp.109.076935

[7] Joukamaa M Heliövaara H Knekt P Aromaa A Raitasalo R Lehtinen V Schizophrenia, neuroleptic medication and mortality. Br J Psychiatry 2006; 188: 122–7. 16449697 10.1192/bjp.188.2.122

[8] Kendrick T Cardiovascular and respiratory risk factors and symptoms among general practice patients with long-term mental illness. Br J Psychiatry 1996; 169: 733–9. 8968631 10.1192/bjp.169.6.733

[9] Brown S Barraclough B Inskip H Causes of the excess mortality of schizophrenia. Br J Psychiatry 2000; 177: 212–7. 11040880 10.1192/bjp.177.3.212

[10] Lawrence DM Holman CD’ AJ Jablensky AV Hobbs MST Death rate from ischaemic heart disease in Western Australian psychiatric patients 1980–1998. Br J Psychiatry 2003; 182: 31–6. 12509315 10.1192/bjp.182.1.31

[11] Marder S Essock S Miller A Buchanan R Casey D David J Physical health monitoring of patients with schizophrenia. Am J Psychiatry 2004; 161: 1334–49. 15285957 10.1176/appi.ajp.161.8.1334

[12] Hennekens C Hennekens A Hollar D Casey D Schizophrenia and increased risks of cardiovascular disease. Am Heart J 2005; 150: 1115–21. 16338246 10.1016/j.ahj.2005.02.007

[13] Gubbins A Lally J McDonald C Metabolic syndrome in patients attending psychiatric day centres: prevalence and associations. Psychiatrist 2012; 36: 326–31.

[14] Lawrence D Hancock K Kisley S The gap in life expectancy from preventable physical illness in psychiatric patients in Western Australia: retrospective analysis of population based registers. BMJ 2013; 346: f2539. 23694688 10.1136/bmj.f2539 PMC3660620

[15] National Institute for Health and Care Excellence. *Lipid Modification: Cardiovascular Risk Assessment and the Modification of Blood Lipids for the Primary and Secondary Prevention of Cardiovascular Disease* (Clinical Guideline CG67). NICE, 2008 (www.nice.org.uk/CG67). 25340243

[16] National Institute for Health and Care Excellence. *Schizophrenia: Core Interventions in the Treatment and Management of Schizophrenia in Primary and Secondary Care* (Clinical Guideline CG1). NICE, 2002.

[17] National Institute for Health and Care Excellence. *Schizophrenia (Update)* (CG82). NICE, 2009

[18] Health and Social Care Information Centre. Quality and Outcomes Framework: GP practice results 2012/13. HSCIC, 2013 (http://www.qof.hscic.gov.uk/index.asp).

[19] Dalton A Soljak M Samarasundera E Millet C Majeed A Prevalence of cardiovascular disease risk amongst the population eligible for the NHS health Check programme. Eur J Prev Cardiol 2013; 20: 142–50. 22058079 10.1177/1741826711428797

[20] Health and Social Care Information Centre. Mental Health Bulletin: Annual Report from MHMDS returns – England 2011–12, initial national figures. HSCIC, 2013: 33–42.

[21] Citrome L Yeomans D Do guidelines for severe mental illness promote physical health and well-being? J Psychopharmacol 2005; 19 (suppl 6): 102–9. 16280343 10.1177/0269881105059505

[22] De Hert M Cohen D Bobes J Cetkovichbakamas M Leucht S Ndetei D Physical illness in patients with severe mental disorder. II. Barriers to care, monitoring and treatment guidelines, plus recommendations at the system and individual level. World Psychiatry 2011; 10: 138–51. 21633691 10.1002/j.2051-5545.2011.tb00036.x PMC3104888

[23] Curtis J Newall H Samaras K Positive Cardiometabolic Health Algorithm 2010. HETI, http://www.heti.nsw.gov.au/cmalgorithm

[24] Rethink. Physical Health Check 2012 (http://www.rethink.org/media/511734/physical_health_check_tool_2013.pdf).

[25] Schizophrenia Commission. The Abandoned Illness: A Report by the Schizophrenia Commission. Schizophrenia Commission, 2012 (http://www.schizophreniacommission.org.uk/).

[26] NHS England. Commissioning for Quality and Innovation (CQUIN): 2014/15 Guidance. NHS England, 2013.

[27] Smith S Yeomans D Bushe C Eriksson C Harrison T Holmes R A well-being programme in severe mental illness. Reducing risk for physical ill-health: a post-programme service evaluation at 2 years. Eur Psychiatry 2007; 22: 413–8. 17765483 10.1016/j.eurpsy.2007.07.002

